# Employment status and cardiometabolic multimorbidity: results from China health and retirement longitudinal study

**DOI:** 10.1186/s12963-026-00459-4

**Published:** 2026-02-02

**Authors:** Yuwei Pan, Martin Bobak, Hynek Pikhart, Jitka Pikhartova

**Affiliations:** Research Department of Epidemiology and Public Health, University College London, 1-19 Torrington Place, London, WC1E 7HB UK

**Keywords:** Cardiometabolic risk factors, Multimorbidity, Employment status, Socioeconomic factors, Longitudinal studies

## Abstract

Globally, cardiometabolic diseases (CMDs) are major health issues that affect the health of workforce. This study aimed to investigate the impact of employment status on transition from a healthy state to cardiometabolic multimorbidity in Chinese population. Data from China Health and Retirement Longitudinal Study (2011–2020) was utilised. Analytical sample comprised 7,681 men and women (≥ 45 years) free of CMDs at baseline. A multistate model was applied to investigate the impact of baseline employment status on the transition rates from a healthy state to cardiometabolic mono-morbidity and subsequently to multimorbidity. Inverse probability weighting was applied to account for the complex survey design. During an average follow-up time of 5.7 years, 3,324 (43.28%) participants developed one or more CMDs. After adjusting for age and sex, compared to non-agricultural employees, non-agricultural retirees had significantly higher risks and agricultural self-employed workers had only marginally higher risk of CMDs. After further adjustment for sociodemographic factors, health behaviours, and BMI, non-agricultural retirees remained significantly associated with a higher rate of transition from a healthy state to cardiometabolic mono-morbidity [HR 1.24 (95% CI 1.01–1.54)] compared to non-agricultural employees. There was no statistically significant increase in transition to multimorbidity risk in any group. Control of CMDs in Chinese older population should consider people’s employment characteristics.

**Supplementary Information:**

The online version contains supplementary material available at 10.1186/s12963-026-00459-4.

## Introduction

Cardiometabolic disease (CMD) refers to an interrelated constellation of diseases such as hypertension, diabetes, dyslipidaemia, coronary heart disease, and stroke [1]. Cardiometabolic multimorbidity, which is defined as coexistence of two or more CMDs, is increasingly common in the Chinese population, with a standardised prevalence of 4.69% among people aged 40 years or over [2].

The current CMD prevention in China focused on traditional risk factors such as diet and beverage, physical activity, smoking, alcohol consumption, and other lifestyle factors [1]. However, apart from these groups of risk factors, the risk of CMD is strongly influenced by socio-economic factors, including factors related to work and employment [3–12]. Existing evidence, mostly from developed countries, showed an increased risk of CMDs associated with employment status, such as unemployment or unstable employment [3, 4], and being housewives [5, 6]. Findings about the effect of self-employment [7, 8], non-standard employment such as part-time jobs [9, 10], and retirement on CMDs were conflicting [11, 12], which may due to the differences in study population, study design, and the varying definitions of the employment characteristics.

In contrast to countries where most of the previous studies on employment and CMDs were conducted, China has been undergoing rapid population ageing, industrialisation, and urbanisation simultaneously over the last few decades. On the one hand, the working-age population has been shrinking [13], while on the other hand, there has been a rapid labour force transition from agricultural industry to non-agricultural sectors [14]. Nevertheless, primary industry workers (refers to agriculture, forestry, animal husbandry, and fishery industries) still account for 22% (163 million) of the total working population in 2024 in China [15]. Despite of the large number of agricultural workers and the urban-rural health inequality, existing studies in China focused on the health effects of employment status among non-agricultural workers due to the lack of statutory retirement age for farmers [16]. Therefore, to assess the potential effect of employment status on CMDs in China’s unique context, especially among agricultural workers, this study will investigate the impact of employment status on the transition from a healthy state to cardiometabolic mono- and subsequently to multimorbidity among Chinese agricultural and non-agricultural workers, which allows for a detailed understanding of the impact of employment status on the onset of a single CMD and the progression from a single CMD to multiple CMDs. Given the urban-rural gap, we hypothesised that agricultural workers and retirees would have higher rates of transition to cardiometabolic mono- and multimorbidity compared to non-agricultural employees.

## Materials and methods

### Study design

Individual-level data from the China Health and Retirement Longitudinal Study (CHARLS) waves 1 to 5 (2011–2020) were utilised. CHARLS is a nationally representative survey of Chinese community residents [17]. It aims to provide longitudinal data covering both health measures and indicators of socio-economic status (SES) of the middle-aged and older adults for the research of Chinese ageing problems [17]. The national baseline survey was conducted between 2011 and 2012 in 28 provinces in China. Primary sampling units (rural villages or urban neighbourhoods) were selected within each county using probabilities proportional to size (PPS) sampling. The overall response rate of main interviews in CHARLS baseline wave was 80.51%, with 17,708 participants successfully interviewed [18]. Participants were intended to be followed up biennially (face-to-face interview). Ethical approval of CHARLS was granted from the Institutional Review Board (IRB) at Peking University [18]. Written informed consent was obtained from each study participant.

### Analytical sample

To reduce reverse causality, participants with any CMD at baseline were excluded, leaving 9,460 participants (main respondents and spouses) who aged 45 years or over had valid information on employment status and were free of CMDs at baseline. An additional 839 participants who lost to follow-up or had missing data in follow-up CMDs were excluded. Since the proportion of missing data in baseline characteristics was about 10%, multiple imputation (for covariates) had little advantage over complete case analysis (CCA) [19, 20]. Therefore, 940 participants were further excluded. Analytical sample comprised 7,681 participants. Supplementary Figure A.1 illustrates the sample selection procedure.

### Employment status

At baseline, CHARLS participants were asked to describe their non-agricultural jobs as earning a wage, running their own business, or working for unpaid family business. According to their responses, participants were categorised into non-agriculturally employed, non-agriculturally self-employed, or working for an unpaid family business. Non-agricultural retirement was defined as retirement (including early retirement and internal retirement) from government departments, enterprises, or institutions. Agricultural employment was defined as working for other farmers in wage for at least 10 days in the past year; agricultural self-employment was defined as doing agricultural work for one’s own household for at least 10 days in the past year; and agricultural retirement was defined as having worked for at least three months during one’s lifetime but did not engage in agricultural work for over 10 days in the past year, nor doing non-agricultural work for at least one hour in the last week, nor on leave, nor searching for a new job in the last month. The definition of employment status referred to the definition of labour force status in harmonised CHARLS (version D) [21]. The groups of non-agricultural unpaid family business and non-agricultural self-employment group were combined due to its small sample size. As a result, employment status was categorised into six groups, including: (1) non-agriculturally employed (reference group), (2) non-agriculturally self-employed or working for an unpaid family business, (3) non-agriculturally retired, (4) agriculturally employed, (5) agriculturally self-employed, and (6) agriculturally retired. Unemployed persons were excluded from the analysis due to their small sample number (*n* = 50).

### Cardiometabolic mono-morbidity and Multimorbidity

Doctor-diagnosed chronic diseases were self-reported in CHARLS interviews. From wave 2 onwards, a question on whether participants had newly diagnosed chronic diseases since the last interview was asked among those who responded to the previous wave. Based on those questions, the following conditions were included in the definition of cardiometabolic mono-morbidity: hypertension, dyslipidaemia, diabetes or high blood sugar, heart attack or other heart problems, and stroke. Cardiometabolic multimorbidity was defined as the coexistence of at least two of the above diseases.

### Covariates

Based on existing evidence from Chinese studies, risk factors of CMDs included older age, sex (female), SES, residence (urban/rural), physically inactive, unhealthy diet, tobacco use, alcohol consumption, lack of sleep, and obesity [1, 22]. Given the data availability in CHARLS and the unique household registration system in China, sociodemographic factors in the current analysis included age, sex, education, yearly household income (quartiles), marital status, household registration status, and regions. Based on the official household registration (also known as Hukou), migrants were defined as rural residents with non-agricultural Hukou or urban residents with agricultural Hukou. Health behaviours included smoking, alcohol consumption, and physical activity (when available). Another important covariate included BMI. BMI was calculated using weight and height measured in the CHARLS physical examination among all participants. However, physical activity was only assessed among a subsample of about half of the households. Therefore, around half of the eligible participants had missing data in physical activity. To retain the sample size at a reasonable level, we only included BMI in the primary analysis and used physical activity for sensitivity analyses. According to the Chinese BMI classification, below 18.5 kg/m^2^ is underweight, 18.5–23.9 kg/m^2^ is normal weight, 24–27.9 kg/m^2^ is overweight, and 28 kg/m^2^ or over is obesity [23, 24].

### Statistical methods

**Fig. 1:**
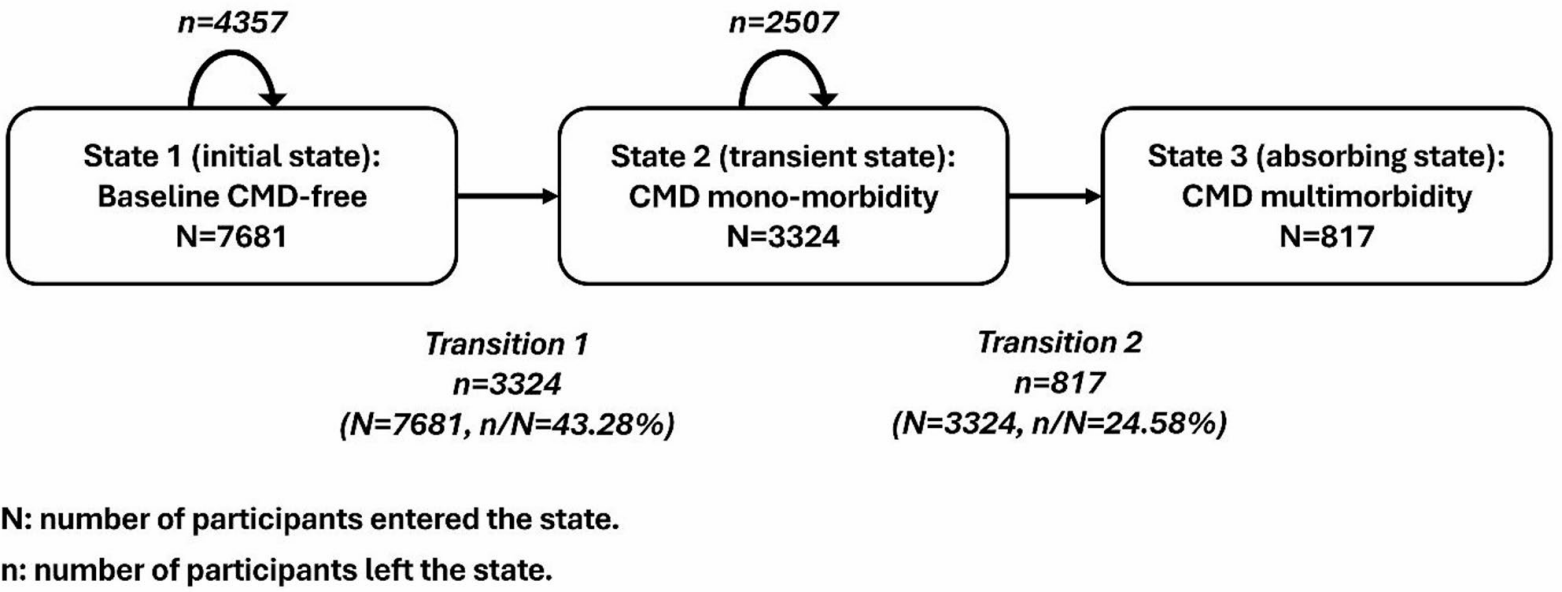
The CMD progressive three-state model, China Health and Retirement Longitudinal Study 2011–2020

Figure 1 illustrates a progressive three-state model (a special case of the $\:k$-progressive model) along with the number of events. State 1 (initial state) is free of CMDs at baseline. Participants may then develop a single CMD (state 2, transient state) and multiple CMD events (state 3, absorbing state). In principle, all participants who developed multiple CMDs should have the stage of one CMD (mono-morbidity) only. However, since in our data only the stage of reporting morbidity is available but not the exact date of CMD diagnosis, for the multimorbidity patients, we assumed that the first CMD event could happen anytime between entry of the study and the stage of reporting the second CMD event and imputed time of the first CMD event. Then we assumed that the second CMD could happen anytime between the imputed time of the first CMD and the stage of reporting the second CMD and imputed time of the second CMD. For the mono-morbidity patients, we assumed that the first CMD could happen anytime between entry of the study and the stage of reporting the first CMD and imputed time of the first CMD. Therefore, among 7,681 participants in the analytical sample, 4,357 participants did not develop any CMD during the follow-up and remained in state 1, 3,324 participants transitioned from a healthy state (state 1) to mono-morbidity (state 2), and 817 participants transitioned from state 2 to multimorbidity (state 3).

A multi-state model (here the $\:k$-progressive model) is often assumed as a Markov model [25]. The Markov assumption states that the future evolution only depends on the event history through the current state [25, 26]. When measuring the time scale, two approaches are frequently used, including ‘clock forward’ and ‘clock reset’ approaches. In a ‘clock forward’ approach, time refers to the time since the patient entered the initial state [26]. In the current study, a ‘clock-forward’ approach was used. Markov stratified hazards models were estimated to investigate the impact of employment status on transition rates from healthy to cardiometabolic mono-morbidity, as well as the transition from cardiometabolic mono- to multimorbidity. Assuming the covariate effects were different for each transition, Cox proportional hazards models were fitted in each transition separately. Participants who remained CMD-free by the end of follow-up were right censored (assumed non-informative censoring). Proportional hazards assumption was tested using Schoenfeld residuals. Covariates were added to the model using a forward stepwise strategy. Interaction terms between employment status and covariates were used to identify effect modification. Wald test was used to assess the joint significance. To account for the complex survey design and loss to follow-up, results were adjusted for individual sample weights.

Since the many persons transitioned from a healthy state (free of CMDs) directly to cardiometabolic multimorbidity (with imputed time of the onset of mono-morbidity), we also conducted a sensitivity analysis, with Cox model to examine the impact of employment status on the transition from baseline healthy status to any cardiometabolic morbidity (mono- or multimorbidity). All the analyses were performed using StataNow/MP 18.5.

## Results

Table 1 shows the sample characteristics at baseline. In Table 1, non-agricultural employees make up 19.55%, while non-agriculturally self-employed workers and unpaid family business workers constitute 10.04%. Agricultural employees account for the smallest proportion, which is 4.14%, while agriculturally self-employed workers account for the largest proportion, which is 42.95%. Non-agricultural and agricultural retirees account for 6.97% and 16.35% of the sample, respectively. During an average follow-up time of 5.7 years, 3,324 (43.28%) participants developed one or more CMDs and 4,357 (56.72%) participants remained free of CMDs. Overall, CMD patients and participants who remained free of CMDs were statistically different in terms of age, sex, household income, region, BMI, and employment status. Supplementary Table A.1 shows the baseline sample characteristics including physical activity.

**Table 1:** Unweighted baseline sample characteristics, China health and retirement longitudinal study 2011–2020

	Total *n* (col %)	Healthy (row %)	CMD cases (row %)	*P*-value^a^
	*N* = 7681	*n* = 4357 (56.72)	*n* = 3324 (43.28)	
**Age**				
45–54	3084 (40.15)	1862 (60.38)	1222 (39.62)	< 0.001^a^
55–64	2907 (37.85)	1556 (53.53)	1351 (46.47)	
65 or over	1690 (22.00)	939 (55.56)	751 (44.44)	
**Sex**				
Female	3733 (48.60)	2044 (54.75)	1689 (45.25)	0.001
Male	3948 (51.40)	2313 (58.59)	1635 (41.41)	
**Education**				
High school or above	835 (10.87)	498 (59.64)	337 (40.36)	0.18^a^
Middle school	1646 (21.43)	927 (56.32)	719 (43.68)	
Elementary school	1702 (22.16)	967 (56.82)	735 (43.18)	
Illiterate	3498 (45.54)	1965 (56.17)	1533 (43.83)	
**Household income**				
1 (highest)	1894 (24.66)	1110 (58.61)	784 (41.39)	0.002^a^
2	1922 (25.02)	1116 (58.06)	806 (41.94)	
3	2020 (26.30)	1140 (56.44)	880 (43.56)	
4 (lowest)	1845 (24.02)	991 (53.71)	854 (46.29)	
**Marital status**				
Married	6874 (89.49)	3883 (56.49)	2991 (43.51)	0.22
Unmarried	807 (10.51)	474 (58.74)	333 (41.26)	
**Household registration status**				
Urban	1064 (13.85)	637 (59.87)	427 (40.13)	0.06
Migrant	1720 (22.39)	954 (55.47)	766 (44.53)	
Rural	4897 (63.75)	2766 (56.48)	2131 (43.52)	
**Regions**				
East	2179 (28.37)	1181 (54.20)	998 (45.80)	< 0.001
Central	1817 (23.66)	974 (53.60)	843 (46.40)	
West	2990 (38.93)	1751 (58.56)	1239 (41.44)	
Northeast	695 (9.05)	451 (64.89)	244 (35.11)	
**Smoking status**				
Never smoker	4391 (57.17)	2454 (55.89)	1937 (44.11)	0.09
Former/current smoker	3290 (42.83)	1903 (57.84)	1387 (42.16)	
**Alcohol consumption**				
Not at all	4,822 (62.78)	2731 (56.64)	2091 (43.36)	0.84
Occasional/frequent drinker	2,859 (37.22)	1626 (56.87)	1233 (43.13)	
**BMI**				
Underweight	577 (7.51)	375 (64.99)	202 (35.01)	< 0.001^a^
Normal	4554 (59.29)	2763 (60.67)	1791 (39.33)	
Overweight	1999 (26.03)	989 (49.47)	1010 (50.53)	
Obesity	551 (7.17)	230 (41.74)	321 (58.26)	
**Employment status**				
Non-agriculturally employed	1502 (19.55)	926 (61.65)	576 (38.35)	< 0.001
Non-agriculturally self-employed	771 (10.04)	440 (57.07)	331 (42.93)	
Non-agriculturally retired	535 (6.97)	288 (53.83)	247 (46.17)	
Agriculturally employed	318 (4.14)	174 (54.72)	144 (45.28)	
Agriculturally self-employed	3299 (42.95)	1824 (55.29)	1475 (44.71)	
Agriculturally retired	1256 (16.35)	705 (56.13)	551 (43.87)	

^a^ Chi-squared for trend where appropriate

**Fig. 2:**
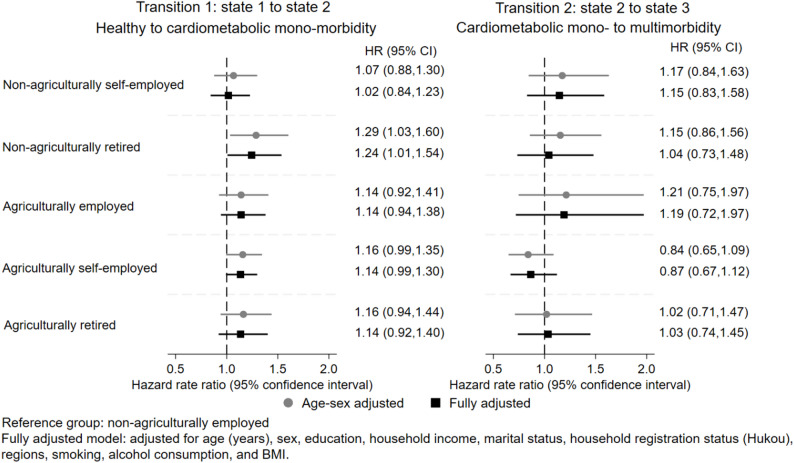
Hazard rate ratio and 95% confidence intervals for all transitions by employment status, China Health and Retirement Longitudinal Study 2011–2020

Figure 2 and supplementary Table A.2 present estimated hazard rate ratios (HRs) with corresponding 95% confidence intervals (CIs) of employment status for all transitions. The global test of Schoenfeld residuals (p-values > 0.05) indicated that the proportional hazards assumption was satisfied. In the age- and sex-adjusted model, compared to non-agricultural employees, agriculturally self-employed workers had 1.16 times the rate of transition from a healthy state to cardiometabolic mono-morbidity (*p* = 0.06), while non-agricultural retirees had 1.29 times the rate of transition from healthy to cardiometabolic mono-morbidity (*p* = 0.02). When controlling for sociodemographic factors, health behaviours, and BMI, non-agricultural retirees remained significantly associated with higher rates of transition from healthy to cardiometabolic mono-morbidity. There was no statistically significant increase in transition to multimorbidity risk in any group. Supplementary Table A.3 shows results additionally adjusted for physical activity.

To validate the robustness of the primary findings, a sensitivity analysis using a binary outcome (any CMD morbidity) via a standard Cox model was conducted. Table 2 shows results of this sensitivity analysis. In Table 2, the effects of employment status were almost identical to its effect in transition one in Fig. 2.

**Table 2:** Hazard rate ratios and 95% confidence intervals of the association between employment status and any cardiometabolic (mono- and multi-) morbidity, China health and retirement longitudinal study 2011–2020

Employment status	Healthy to CMD mono-/multi-morbidity				
	CMD/N	Age-sex adjusted		Fully adjusted^a^	
	3324/7681	HR (95% CI)	p-value	HR (95% CI)	p-value
Non-agriculturally employed	576/1502	Ref		Ref	
Non-agriculturally self-employed	331/771	1.07 (0.88, 1.30)	0.51	1.02 (0.84, 1.23)	0.83
Non-agriculturally retired	247/535	1.26 (0.98, 1.61)	0.06	1.23 (0.98, 1.54)	0.07
Agriculturally employed	144/318	1.14 (0.92, 1.41)	0.23	1.15 (0.95, 1.39)	0.17
Agriculturally self-employed	1475/3299	1.15 (0.98, 1.34)	0.10	1.13 (0.98, 1.30)	0.09
Agriculturally retired	551/1256	1.16 (0.93, 1.45)	0.18	1.14 (0.92, 1.41)	0.23

CMD: number of CMD cases in the group; N: Number of participants in the group

^a^Fully adjusted model: adjusted for age (continuous), sex, education, household income, marital status, household registration status (Hukou), regions, smoking, alcohol consumption, and BMI

## Discussion

### Main findings of this research

Among Chinese middle-aged and older population, compared to non-agricultural employees, non-agricultural retirees had a significantly higher rate of transition from a healthy state to cardiometabolic mono-morbidity but not subsequent multimorbidity. There is insufficient evidence of increased risk of CMDs among other groups of workers.

### Comparison with existing studies

Previous findings on employment status and self-rated health (SRH) showed that Chinese non-agricultural employees had considerably better health status than other working groups among middle-aged and older population [27]. The current results further confirmed this finding using objective health outcomes - CMDs.

In the framework of social determinants of health proposed by Marmot and Wilkinson, work is one of the main pathways linking social structure to health and well-being [28]. Over the last few decades, as a result of the rapid social and economic development in China, working conditions changed dramatically. Along with a decreased proportion of working age population (15 to 64 years old), China has large numbers of agricultural workers and rural migrant workers [13, 15]. According to the National Bureau of Statistics (NBS) of China, 163 million people worked in the primary industry in 2024 [15], and the number of rural migrant workers in that year was 300 million [14]. This trend of nationwide labour force transition from agricultural to non-agricultural sectors in China may has an impact on the population health. Previous studies showed that rural agricultural workers had a lower prevalence of hypertension, overweight, and obesity than urban non-agricultural workers [29]. However, current results indicated that agriculturally self-employed workers (the majority of them are rural residents) had marginally higher rates of transition from a healthy state to cardiometabolic mono-morbidity than non-agricultural employees. Possible reasons include limited access to healthcare resources in rural areas, a lower standard of professionalism of some doctors in rural areas, and the low household income of some farmers [30].

Non-agricultural self-employment was an important element of the transition from agricultural to non-agricultural sectors in China. Results of the Chinese Household Income Project 1988–2018 showed that adults with self-employment as primary off-farm job in rural China increased from 2% in 1988 to 12% in 2013 [31]. This increase halted between 2013 and 2018, probably due to the out-migration of young labour force [32, 33], the drop in birth rate, and new policies benefiting farmers. Previous studies from other populations reported conflicting findings on the effect of self-employment on CMDs [7, 8]. In the current Chinese middle-aged and older population, we did not find enough evidence for the increased risk of CMDs among non-agriculturally self-employed workers compared with non-agricultural employees. The different social context in China and other countries might contribute to the different findings.

Existing evidence on the effect of retirement on CMDs and its risk factors was contradictory [11, 12]. The current results showed that non-agricultural retirement was associated with increased rates of transitioning from a healthy state to cardiometabolic mono-morbidity, independently from age and other risk factors, which was consistent with some of the previous findings. For example, evidence from the Health and Retirement Study (HRS) showed participants who were fully retired had 1.40 times higher risk of CVD onset compared with full-time workers after full adjustment [34]. Results from CHARLS 2011–2015 showed that non-agricultural retirement was associated with higher weight and BMI, which were important risk factors of CMDs, although this effect was only observed in men [35]. Meanwhile, a previous study involving participants from 35 countries found that, although retirement was associated with a decreased risk of heart disease on average [12], its associations with stroke and obesity were heterogeneous by individual characteristics including education and pre-retirement job [12]. Overall, factors such as the different definitions of retirement, years after retirement, pre-retirement occupation and health, may all contribute to the different findings on the association between retirement and CMDs.

### Strengths and limitations

First, this research used nationally representative data from a prospective cohort study. Therefore, the temporal sequence is clear. Second, instead of studying CMDs with combined mono- and multimorbidity, this research used a multistate model to investigate the effect of employment status on the transition from a healthy state to cardiometabolic mono-morbidity and ultimately to multimorbidity, which allowed for a detailed understanding of the impact of employment on the onset of a single CMD and the progression from a single CMD to multiple CMDs.

However, there are still several limitations to be considered. First, the employment information was from baseline wave. Therefore, the time-varying effect of employment status such as the transition into retirement was not investigated. Second, the current research used doctor-diagnosed self-reported CMDs as the outcome measurement. Although information was confirmed in the following wave, potential recall bias and misclassification could not be excluded. This may result in underestimation of the association if less CMD events were reported than in the general population if the recall was imprecise. Third, the lack of data on the exact date of onset of mono- and multi-morbidity may have introduced some imprecision in the estimation of the separate effects of employment status on mono- vs. multi-morbidity. However, this issue would not affect the estimation of the overall effects on the combined (mono- and multi-) morbidity (Table 2). Fourth, although participants with CMDs at baseline were excluded, reverse causality may still be present as agricultural workers might opt to continue working until their health conditions preclude further labour.

## Conclusions

Chinese non-agricultural retirees had higher risks of CMDs than non-agricultural employees, and agriculturally self-employed workers had marginally increased risks of CMDs. CMD control among middle-aged and older population in China should take into account the employment characteristics of this population.

## Supplementary Information

Supplementary Material 1.

## Data Availability

The datasets supporting the conclusions of this article are available from China Health and Retirement Longitudinal Study project home page, [https://charls.pku.edu.cn/en/index.htm](https:/charls.pku.edu.cn/en/index.htm).
