# Supplementary material for: Employment status and cardiometabolic multimorbidity: results from China health and retirement longitudinal study
Source: Popul Health Metr. 2026 Feb 2;24:14. doi: 10.1186/s12963-026-00459-4 (PMC12955182; doi:10.1186/s12963-026-00459-4)
Supplement: Supplementary file 1 — Supplementary Material 1. [file 12963_2026_459_MOESM1_ESM.docx]

**Appendix A**

**Supplementary materials**

**Figure A.1** Sample selection procedure of the analyses on employment status and cardiometabolic diseases (CMDs), China Health and Retirement Longitudinal Study (CHARLS) 2011-2020

Participants who were CMDs-free at baseline

n=10370

CHARLS wave 1 participants

n=17708

Age-eligible participants (≥45 years old)

n=17319

Participants with information on employment status

n=9460

Analytical sample

n=7681

Participants with follow-up information on CMDs

n=8621

n=389 participants missing age or aged under 45

n=435 missing baseline information on CMDs

n=6514 reported CMDs at baseline

n=910 without valid employment information in w1

n=839 missing follow-up information on CMDs in w2-5

n=940 missing information on baseline characteristics

**Table A.1** Unweighted baseline sample characteristics, China Health and Retirement Longitudinal Study 2011-2020 (additionally including physical activity, n=3142)

|  | Total n  (col %) | Healthy (row %) | CMD cases (row %) | P-value^a^ |
| --- | --- | --- | --- | --- |
|  | N=3142 | n=1796  (57.16) | n=1346  (42.84) |  |
| **Age** |  |  |  |  |
| 45-54 | 1,284 (40.87) | 767 (59.74) | 517 (40.26) | 0.004 |
| 55-64 | 1,182 (37.62) | 631 (53.38) | 551 (46.62) |  |
| 65 or over | 676 (21.51) | 398 (58.88) | 278 (41.12) |  |
| **Sex** |  |  |  |  |
| Female | 1,582 (50.35) | 870 (54.99) | 712 (45.01) | 0.01 |
| Male | 1,560 (49.65) | 926 (59.36) | 634 (40.64) |  |
| **Education** |  |  |  |  |
| High school or above | 340 (10.82) | 201 (59.12) | 139 (40.88) | 0.44 |
| Middle school | 677 (21.55) | 369 (54.51) | 308 (45.49) |  |
| Elementary school | 681 (21.67) | 393 (57.71) | 288 (42.29) |  |
| Illiterate | 1444 (45.96) | 833 (57.69) | 611 (42.31) |  |
| **Household income** |  |  |  |  |
| 1 (highest) | 786 (25.02) | 455 (57.89) | 331 (42.11) | 0.21 |
| 2 | 794 (25.27) | 450 (56.68) | 344 (43.32) |  |
| 3 | 843 (26.83) | 501 (59.43) | 342 (40.57) |  |
| 4 (lowest) | 719 (22.88) | 390 (54.24) | 329 (45.76) |  |
| **Marital status** |  |  |  |  |
| Married | 2,821 (89.78) | 1,599 (56.68) | 1222 (43.32) | 0.11 |
| Unmarried | 321 (10.22) | 197 (61.37) | 124 (38.63) |  |
| **Household registration status** |  |  |  |  |
| Urban | 426 (13.56) | 258 (60.56) | 168 (39.44) | 0.31 |
| Migrant | 712 (22.66) | 405 (56.88) | 307 (43.12) |  |
| Rural | 2,004 (63.78) | 1,133 (56.54) | 871 (43.46) |  |
| **Regions** |  |  |  |  |
| East | 871 (27.72) | 490 (56.26) | 381 (43.74) | 0.01 |
| Central | 740 (23.55) | 390 (52.70) | 350 (47.30) |  |
| West | 1,238 (39.40) | 733 (59.21) | 505 (40.79) |  |
| Northeast | 293 (9.33) | 183 (62.46) | 110 (37.54) |  |
| **Smoking status** |  |  |  |  |
| Never smoker | 1,885 (59.99) | 1,072 (56.87) | 813 (43.13) | 0.69 |
| Former/current smoker | 1,257 (40.01) | 724 (57.60) | 533 (42.40) |  |
| **Alcohol consumption** |  |  |  |  |
| Not at all | 1,994 (63.46) | 1,140 (57.17) | 854 (42.83) | 0.99 |
| Occasional/frequent drinker | 1,148 (36.54) | 656 (57.14) | 492 (42.86) |  |
| **BMI** |  |  |  |  |
| Underweight | 232 (7.38) | 149 (64.22) | 83 (35.78) | <0.001^a^ |
| Normal | 1,862 (59.26) | 1,151 (61.82) | 711 (38.18) |  |
| Overweight | 830 (26.42) | 411 (49.52) | 419 (50.48) |  |
| Obesity | 218 (6.94) | 85 (38.99) | 133 (61.01) |  |
| **Physical activity^b^** |  |  |  |  |
| Vigorous (≥1 time/week) | 1,326 (42.20) | 773 (58.30) | 553 (41.70) | 0.30 |
| Moderate (≥1 time/week) | 949 (30.20) | 528 (55.64) | 421 (44.36) |  |
| Walking (≥1 time/week) | 603 (19.19) | 354 (58.71) | 249 (41.29) |  |
| No physical activity | 264 (8.40) | 141 (53.41) | 123 (46.59) |  |
| **Employment status** |  |  |  |  |
| Non-agriculturally employed | 567 (18.05) | 346 (61.02) | 221 (38.98) | 0.10 |
| Non-agriculturally self-employed | 325 (10.34) | 187 (57.54) | 138 (42.46) |  |
| Non-agriculturally retired | 210 (6.68) | 106 (50.48) | 104 (49.52) |  |
| Agriculturally employed | 127 (4.04) | 68 (53.54) | 59 (46.46) |  |
| Agriculturally self-employed | 1,400 (44.56) | 786 (56.14) | 614 (43.86) |  |
| Agriculturally retired | 513 (16.33) | 303 (59.06) | 210 (40.94) |  |

^a^ Chi-squared for trend where appropriate.

^b^ Physical activity was measured by three sets of questions in the CHARLS Health status and functioning section in waves 1 to 4. Questions on the frequency and duration of vigorous activity, moderate physical activity, and walking were asked, such as ‘During a usual week, did you do any [vigorous activity; moderate physical activity; or walking] for at least 10 minutes continuously?’ Vigorous activity was defined as activity that make you breathe much harder than normal and may include heavy lifting, digging, plowing, aerobics, fast bicycling, and cycling with a heavy load. Moderate physical activity was defined as activity that make you breathe somewhat harder than normal and may include carrying light loads, bicycling at a regular pace, or mopping the floor. To reduce missing data, we combined three sets of questions.

**Table A.2** Hazard rate ratio and 95% confidence intervals for all transitions by employment status, China Health and Retirement Longitudinal Study 2011-2020 (n=7681)

|  | Transition 1: state 1 to state 2  (healthy to cardiometabolic mono-morbidity) | | | | Transition 2: state 2 to state 3  (cardiometabolic mono- to multimorbidity) | | | |
| --- | --- | --- | --- | --- | --- | --- | --- | --- |
|  | Age-sex adjusted | | Fully adjusted^a^ | | Age-sex adjusted | | Fully adjusted^a^ | |
| Employment status | HR (95% CI) | P-value | HR (95% CI) | P-value | HR (95% CI) | P-value | HR (95% CI) | P-value |
| Non-agriculturally employed | Ref | - | Ref | - | Ref | - | Ref | - |
| Non-agriculturally self-employed | 1.07 (0.88,1.30) | 0.52 | 1.02 (0.84,1.23) | 0.86 | 1.17 (0.84,1.63) | 0.34 | 1.15 (0.83,1.58) | 0.41 |
| Non-agriculturally retired | 1.29 (1.03,1.60) | 0.02 | 1.24 (1.01,1.54) | 0.04 | 1.15 (0.86,1.56) | 0.34 | 1.04 (0.73,1.48) | 0.82 |
| Agriculturally employed | 1.14 (0.92,1.41) | 0.22 | 1.14 (0.94,1.38) | 0.17 | 1.21 (0.75,1.97) | 0.44 | 1.19 (0.72,1.97) | 0.50 |
| Agriculturally self-employed | 1.16 (0.99,1.35) | 0.06 | 1.14 (0.99,1.30) | 0.06 | 0.84 (0.65,1.09) | 0.19 | 0.87 (0.67,1.12) | 0.27 |
| Agriculturally retired | 1.16 (0.94,1.44) | 0.16 | 1.14 (0.92,1.40) | 0.23 | 1.02 (0.71,1.47) | 0.91 | 1.03 (0.74,1.45) | 0.84 |

^a^ Fully adjusted model: adjusted for age (continuous), sex, education, household income, marital status, household registration status (Hukou), regions, smoking, alcohol consumption, and BMI.

**Table A.3** Hazard rate ratio and 95% confidence intervals for all transitions by employment status (additionally adjusted for physical activity), China Health and Retirement Longitudinal Study 2011-2020 (n=3142)

|  | Transition 1: state 1 to state 2  (healthy to cardiometabolic mono-morbidity) | | | | Transition 2: state 2 to state 3  (cardiometabolic mono- to multimorbidity) | | | |
| --- | --- | --- | --- | --- | --- | --- | --- | --- |
|  | Age-sex adjusted | | Fully adjusted^a^ | | Age-sex adjusted | | Fully adjusted^a^ | |
| Employment status | HR (95% CI) | P-value | HR (95% CI) | P-value | HR (95% CI) | P-value | HR (95% CI) | P-value |
| Non-agriculturally employed | Ref | - | Ref | - | Ref | - | Ref | - |
| Non-agriculturally self-employed | 1.09 (0.80,1.48) | 0.60 | 1.04 (0.76,1.42) | 0.82 | 1.14 (0.64,2.03) | 0.65 | 1.26 (0.75,2.11) | 0.38 |
| Non-agriculturally retired | 1.46 (1.01,2.12) | 0.05 | 1.47 (1.02,2.13) | 0.04 | 1.35 (0.77,2.37) | 0.29 | 1.38 (0.73,2.63) | 0.32 |
| Agriculturally employed | 1.13 (0.84,1.51) | 0.41 | 1.16 (0.86,1.57) | 0.32 | 1.39 (0.61,3.17) | 0.43 | 1.42 (0.59,3.39) | 0.43 |
| Agriculturally self-employed | 1.20 (0.98,1.45) | 0.07 | 1.17 (0.95,1.44) | 0.14 | 0.87 (0.54,1.43) | 0.59 | 0.95 (0.62,1.46) | 0.82 |
| Agriculturally retired | 1.28 (0.88,1.86) | 0.19 | 1.27 (0.92,1.77) | 0.15 | 1.03 (0.58,1.82) | 0.93 | 0.99 (0.58,1.69) | 0.98 |

^a^ Fully adjusted model: adjusted for age (continuous), sex, education, household income, marital status, household registration status (Hukou), regions, smoking, alcohol consumption, BMI, and physical activity.

Notes: Baseline physical activity was only asked among a subsample of the CHARLS households (half). Therefore, only 3142 participants were included this complete case analysis. Among these 3142 participants, 1326 participants reported doing vigorous activity at least once a week, 949 participants reported doing moderate activity at least once a week, 603 participants reported walking at least once a week, and 264 participants reported no physical activity.
